# Stem the blood flow: beneficial impact of bevacizumab on survival of subventricular zone glioblastoma patients

**DOI:** 10.1007/s11060-024-04828-7

**Published:** 2024-09-24

**Authors:** Yosef Laviv, Ohad Regev, Andrew A. Kanner, Susana Fichman, Dror Limon, Tali Siegal, Shlomit Yust-Katz, Alexandra Benouaich-Amiel

**Affiliations:** 1 Neurosurgery department, Beilinson hospital, Rabin Medical Center, 39 Zeev Jabotinsky St, Petach Tikva, 4941492 Israel; 2 Sackler Faculty of Medicine, Tel Aviv University, Tel Aviv, Israel; 3 Pathology department, Beilinson hospital, Rabin Medical Center, Petah Tikva, Israel; 4 Neuro-Oncology Unit, Davidoff Cancer Center, Beilinson Hospital, Rabin Medical Center, Petah Tikva, Israel; 5 Hebrew University, Jerusalem, Israel; 6 Meir Medical Center, Kfar Saba, Israel

**Keywords:** Angiogenesis, Bevacizumab, Glioblastoma, Subventricular zone, Glioma stem cells

## Abstract

**Purpose:**

Angiogenesis is a crucial step in tumorigenesis of glioblastoma (GBM). Bevacizumab, an anti-vascular endothelial growth factor drug, is approved for second-line therapy for GBM. Glioma stem cells, presumably the cell of origin of GBM, take an active role in angiogenesis. The subventricular zone (SVZ) is the brain’s largest reservoir of neural stem cells, and GBM near this region (SVZ GBM) is associated with a poor prognosis. This study aims to evaluate the potential impact of second-line bevacizumab treatment on survival in patients with SVZ GBM.

**Methods:**

The electronic medical records of adult patients with newly diagnosed SVZ GDM under treated between 1/2011 and 12/2021 were retrospectively reviewed. Clinical, surgical, radiological, and outcome parameters were compared between patients treated with bevacizumab after first relapse to patients without such treatment.

**Results:**

The cohort included 67 patients. 45 (67.1%) were treated with bevacizumab after the first relapse while 22 (32.9%) were not. The only statistically significant difference between groups was the rate of re-surgery, which was higher in the *non-*bevacizumab group (40.9% vs. 15.6%; *p* = 0.023), indicating that the groups were quite homogenous. In general, bevacizumab as a second-line treatment did not affect OS in SVZ GBM cases. However, it significantly prolongs survival time from 1st relapse by an average of more than 4 months, including after adjustment to re-surgery variable (HR = 0.57, 95% CI 0.34–0.94, *p* = 0.028 and HR = 0.57, 95%CI = 0.34–0.97, PV = 0.038; respectively). Furthermore, when adjusting to time from diagnosis to 1st relapse, bevacizumab treatment was also associated with prolonged OS (HR = 0.58; *p* = 0.043). In a subgroup analysis, comparing patients treated with both re-surgery and bevacizumab to patients treated in any other way, patients with the combined treatment had the longest mean OS of the entire cohort (22.16 ± 7.81 m vs. 13.60 ± 6.86, *p* = 0.049; HR = 0.361 95%CI 0.108–1.209, *p* = 0.085).

**Conclusions:**

The use of bevacizumab as a second-line therapy in SVZ GBM cases may positively affect survival after relapse, even when given as a monotherapy. Additionally, in certain yet-to-be-identified sub-populations, bevacizumab may even extend overall survival. Further research is required to accurately identify SVZ GBM patients who would benefit most from anti-angiogenic therapy.

## Introduction

Glioblastoma (GBM) is the most malignant primary brain tumor. Median survival is 15–20 months even with intensive treatment that includes maximal safe resection, chemotherapy, and radiation [1]. In 50 − 60% of cases, the tumor involves the outside lining of the lateral cerebral ventricles [2], called the subventricular zone (SVZ). This zone is the largest neural stem cell (NSCs) niche in the adult brain [3]. NSCs play a role in tumorigenesis and angiogenesis [4–6]. Recent studies showed that SVZ involvement is an independent, adverse prognostic factor in GBM [1, 2, 7–9]. SVZ tumors are associated with significantly greater volume at presentation, multifocal tumor growth, a lesser extent of resection (EOR), worse functional postoperative outcome, and shorter overall survival (OS) [10, 11]. Therefore, SVZ GBM should be considered a specific oncological entity, worthy of more extensive characterization [10].

Angiogenesis is a crucial mechanism for tumor cell survival, providing nutrients and oxygen, and promotes tumor immunosuppressive effect [12]. In GBM, the high metabolic demand of tumor cells for oxygen and nutrients often surpasses the available supply, resulting in hypoxia [13]. In turn, this initiates transcription of vascular endothelial growth factor (VEGF) protein, leading to angiogenesis that maintains the tumor’s vascular supply and promotes tumor-cell survival [14]. Highly vascular features of GBMs have been repeatedly demonstrated [15].

Antiangiogenic therapy has been an extensively studied strategy for GBM in the past decade. In this context, the human monoclonal antibody bevacizumab, which targets VEGF, received approval from the Food and Drug Administration (FDA) for the treatment of GBM at first relapse after standard chemoradiation. This approval was granted due to bevacizumab’s ability to prolong progression-free survival (PFS) after 1st recurrence and provide clinical benefits, such as alleviating neurological symptoms [16]. Although bevacizumab did not extend OS in phase 3 clinical trials of newly diagnosed [17, 18] or recurrent GBM [16], these trials did not analyze SVZ GBM as a subgroup, either in the original studies or in subsequent post hoc analyses [19]. Thus, data on the survival impact of bevacizumab in SVZ GBM is lacking.

Given the role of NSCs in angiogenesis and tumorigenesis, we sought to investigate the potential impact of bevacizumab on OS and survival from the first relapse in SVZ GBM. To this end, patients with SVZ GBM were retrospectively divided into two groups: treated or not treated with bevacizumab at first relapse. Groups were compared for different epidemiological, clinical, surgical, radiological, molecular, and survival parameters. Results were statistically analyzed to demonstrate any significant differences between groups.

## Materials and methods

### Design and patients

This study was approved by the Institutional Review Board and conducted in accordance with ethical standards of the 1964 Helsinki Declaration and its later amendments.

**Fig. 1:**
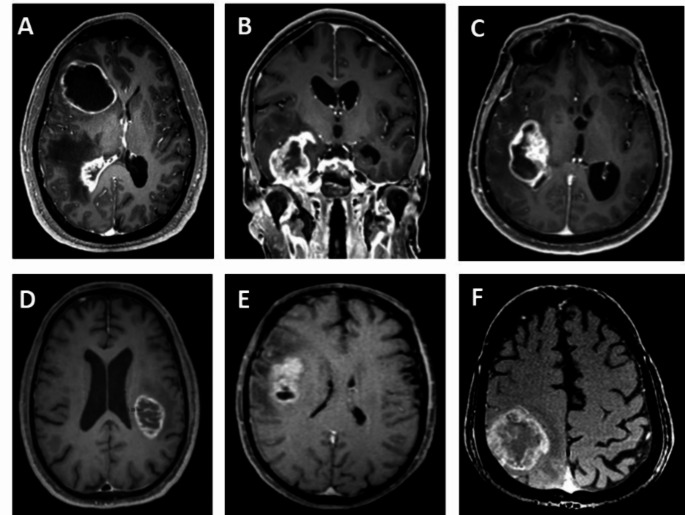
Illustrative cases. Gadolinium-enhanced T1-weighted magnetic resonance imaging. Upper row: SVZ-GBM. (**A**) Lateral ventricle, frontal and occipital horns; axial view. (**B**) Lateral ventricle, temporal horn; coronal view. (**C**) Lateral ventricle, occipital horn; axial view. Lower row: non-SVZ GBM. **A-C**) Increasing distances from the wall of the lateral ventricles; axial views

A retrospective search of the electronic and computerized medical records was performed to identify all adult patients (age ≥ 18 years) with newly diagnosed SVZ GBM treated at a single tertiary medical center between January 2011 and December 2021. Patients fulfilling the following criteria were included in the study: diagnosis of GBM according to WHO classification validated at time of the diagnosis [20–22]; diagnosis of SVZ GBM defined as GBM in direct contact with the walls of the lateral ventricles or situated within 2 mm of the lateral ventricular ependyma on gadolinium-enhanced T1-weighted magnetic resonance imaging (MRI) scans, as previously described [8] (Fig. 1); partial or complete surgical resection at diagnosis; and chemoradiation therapy as first-line treatment.

Exclusion criteria were as follows: a history of low-grade glioma; isocitrate dehydrogenase (IDH) mutations (determined by either immunohistochemistry or next-generation sequencing in order to adhere to the changes in the World Health Organization classification of high-grade gliomas during the period of the study [22]); no documented disease progression during the study period; and insufficient data or loss to follow up.

### Data collection

Demographic, clinical, radiological, surgical, and pathological/molecular data of all eligible patients were extracted from the medical records as follows: age at diagnosis and sex; type and duration of symptoms, findings on neurological examination at presentation, postoperative Karnofsky Performance Scale (KPS) score, length of hospitalization, need for rehabilitation, time to oncological treatment, type of adjuvant therapy, number of temozolomide cycles, need for a second surgery, KPS pre second-line therapy, type of second-line therapy, PFS, time from progression to death or last follow up and OS; preoperative and postoperative tumor volume, the extent of resection, distant recurrence as first relapse; maximum Ki-67 level, maximum TP53 level, and O6-methylguanine-DNA-methyltransferase (*MGMT*) methylation status.

### Group allocation and comparison

All patients underwent surgical intervention for tumor resection, followed by adjuvant chemoradiation (either the Stupp protocol [23] or the “short” protocol [24]), according to clinical decisions made by the neuro-oncologists involved (S.Y., T.S. and A.A.). Based on RANO criteria [25], patients with first disease progression were eligible for second-line treatment which was not protocol-based and included a diversity of options such as temozolomide, radiosurgery, bevacizumab, lomustine, Tumor Treating Fields (Novocure©), and clinical trials of tumor-targeted vaccines and immunotherapy. Patients were then classified as treated or not treated with second-line bevacizumab and compared for the mentioned variables and survival. Bevacizumab (AVASTIN^®^) was administered intravenously at a dose of 10 mg/kg every 2 weeks, as recommended for recurrent GBM. Those who received fewer than two cycles of bevacizumab were classified as “not treated.”

### Outcome measures

Volumetric analysis was performed using Brainlab Smart Brush^®^ software (BrainLAB AG©, Munich, Germany). Calculations of tumor volume were based on 3D reconstructions of the tumor.

All volumetric analyses were conducted on post-gadolinium T1-weighted MRI studies performed within 48 h of surgery. EOR was based on post-operative volumes of contrast-enhancing (CE) and non-CE residual, In accordance with the newly reported RANO categories for extent of resection in glioblastoma [26]: class 1 (supramaximal CE resection): Ocm^3^ CE residual + < 5cm^3^ non-CE residual; class 2 A (maximal, complete CE resection): Ocm^3^ CE residual + > 5cm^3^ non-CE residual; class 2B (maximal, near total CE resection): ≤1cm^3^ CE residual; class 3A (submaximal, subtotal CE resection): ≤5cm^3^ CE residual; class 3B(submaximal, partial CE resection): >5cm^3^ CE residual. Patients who underwent biopsy only (class 4) were excluded. Non-CE volumes was determined by the extent of peritumoral high intensity signal on T2-weighted fluid-attenuated inversion recovery (FLAIR) imaging.

Distant parenchymal recurrences were defined as new contrast-enhancing foci located > 2.0 cm away from the initial tumor borders.

PFS was defined as the time from the date of diagnosis to the first radiological progression. OS was defined as the time from the date of diagnosis to death (non-censored) or last follow-up (censored). Time from first relapse to death (non-censored) or last follow-up (censored) was also documented. Patients who were lost to follow-up were excluded.

### Statistical analysis

Descriptive statistics were employed to analyze the attributes of the study population. Each variable was presented by the most suitable central and dispersion measures: nominal variables were presented by number and percent (%), numerical variables were presented by either mean ± standard deviation (SD) or median and inter-quartile range (IQR). Normal distribution of numerical variables was assessed using histograms, Q-Q plots, Shapiro-Wilk test, and Kolmogorov-Smirnov test.

First, we conducted univariate analysis to assess the clinical and sociodemographic characteristics of the study cohort stratified by bevacizumab administration. For continuous variables we used Man-Whitney test due to their non-normal distribution, and for nominal variables we used either Chi-square test of Fisher exact test. Next, we examined the association between bevacizumab administration to all-cause mortality using Kaplan-Meier curves and Log-rank test. Finally, we used univariate and multivariable Cox regression to assess bevacizumab administration Hazard ratio (HR), after adjusting to the time from diagnosis to 1st recurrence and beginning of bevacizumab administration.

All analyses were conducted using SPSS Statistics (IBM, Armonk, NY, USA; version 28) and R software. A two-sided test significance level of 0.05 was used throughout the entire study.

## Results

**Fig. 2:**
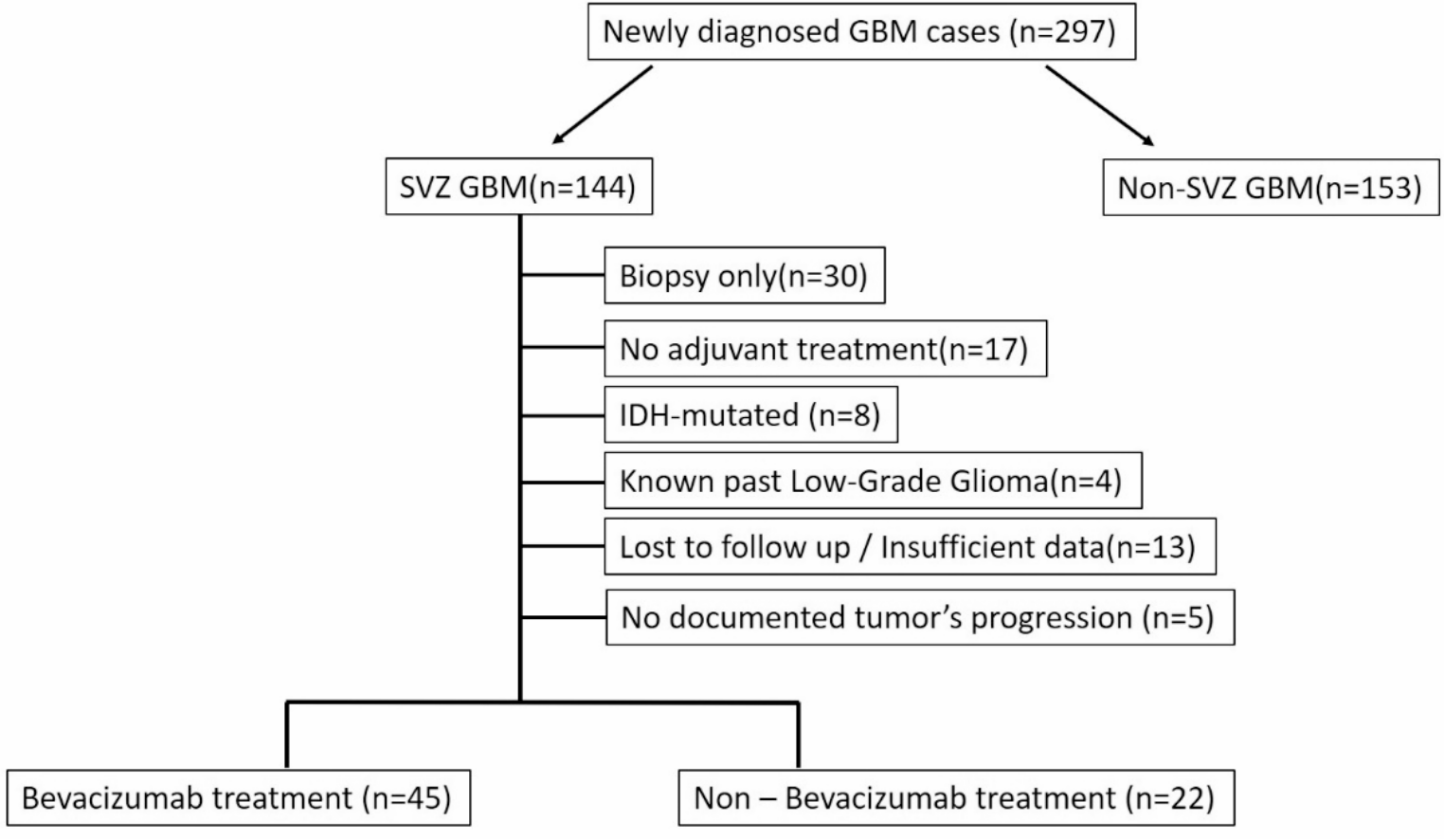
Schematic illustration of study profile

During the study period, 297 patients with newly diagnosed GBM underwent surgical intervention at our institution. Of these, 230 were excluded from the analysis as shown in Fig. 2.

The remaining 67 patients formed the study cohort. They included 34 men and 33 women with a mean age of 58.11 years. The anatomic distribution of the tumors was as follows: 26 temporal, 24 frontal, 22 parietal, and 13 occipital; in 25 cases, more than one lobe was involved. Most patients (> 92%) were treated according to Stupp protocol, with no significant difference between groups. Of the entire cohort, 59 (88.0%) patients have received second-line treatment. Of those, 36 patients were given monotherapy (bevacizumab as a monotherapy in 27 cases), and 23 patients were given combined therapy (bevacizumab as a combined therapy in 18 cases). Together, 45 (67.1%) patients were treated with bevacizumab after first progression (= bevacizumab group) and 22 (32.9%) were not (= non-bevacizumab group). The median bevacizumab cycles in the treated group were 9. Of note, 13 additional patients were offered bevacizumab and either did not receive it (*n* = 9) or were treated with up to 2 cycles (*n* = 4). Other second-line treatment modalities included: Tumor Treating Fields (*n* = 13), temozolomide (*n* = 7), radiation (*n* = 5), lomustine (*n* = 4), pembrolizumab (*n* = 1), everolimus (*n* = 1) and clinical trial (*n* = 1). Of the entire cohort, 16 patients (23.8%) underwent re-surgery before beginning second-line treatment. Among these, six patients received bevacizumab after their surgery.

As shown in Table 1, there were no significant differences between the bevacizumab and non- bevacizumab groups in most of the demographic, clinical, surgical, oncological, and prognostic variables evaluated. The only statistically significant difference between the two groups was the rate of re-surgery, which was higher in the non*-* bevacizumab group (40.9% vs. 15.6%, respectively; *p* = 0.023). The difference in age between groups has reached a near significance.

**Table 1:** Clinical and sociodemographic characteristics of study cohort

Variable	All Patients (*n* = 67)	No Bevacizumab (*N* = 22)	Bevacizumab (*N* = 45)	*P*-value
Age at Diagnosis, years	58.1 ± 14.0	62.6 ± 13.3	55.7 ± 13.0	*0.071*
Gender (M: F)	1.09	1.54	0.86	0.327
Clinical presentation, %				
Increased intracranial pressure	50.7	46.4	53.7	0.628
Cognitive impairment	29	28.6	29.3	1.000
Seizures	2.9	0.0	4.9	0.511
Focal signs or symptoms	50.7	50.0	51.2	1.000
Duration of symptoms (weeks), mean ± SD	3.79 ± 4.17	4.24 ± 5.06	3.12 ± 3.90	0.308
Methylated MGMT, n (%)*	20(47.8)	6(50.0)	14(43.8)	0.709
1st treatment KPS (pre adjuvant treatment)	80(70–90)	80(60–90)	80(70–90)	0.501
Pre-Surgery Volume, cm^3^	43.1 ± 28.7	38.7 ± 18.3	42.8 ± 32.0	0.631
Post-Surgery Volume, cm^3^	5.0 ± 6.1	4.8 ± 4.8	5.3 ± 6.9	0.792
Percent of Resection, %	86.6 ± 15.9	87.4 ± 16.7	86.0 ± 15.6	0.569
RANO categories EOR (%)				
Class 1	6.7	9.5	5,1	0.606
Class 2	26.7	33.3	23.1	0.541
Class 3 A	28.3	19.0	33.3	0.369
Class 3B	38.3	38.1	38.5	1.000
Postoperative home discharge, %	78.2	71.4	82.9	0.373
2nd treatment KPS (pre second line treatment)	70(60–80)	70(55–80)	70(70–80)	0.107
“Stupp” protocol, y (%)	62(92.5)	19(86.3)	43(95.5)	0.273
Temozolomide cycles (n), mean ± SD	3.55 ± 3.42	4.15 ± 3.12	3.06 ± 2.73	0.168
Distant recurrence, %	15.1	15.4	15	1.000
Re-Surgery, %	23.8	40.9	15.6	**0.023**
TTFields at any Time	14(20.8)	4(18.1)	10(22.2)	0.741
Time to Oncology Treatment, weeks	4.5(4–6)	4(4-5.75)	5(4–6)	0.215
Time from Diagnosis until 1st recurrence, months	6.5(3.8–13)	9.0(4.6–14)	6.0(3.5–11)	0.143

Note: Boldface type indicates *p* < 0.05; Italic type indicated near significance

Number (%); Mean ± Standard Deviation; Median (Inter-quartile Range)

* MGMT status was not available for all patients

Table 2 shows the association between bevacizumab and different survival variables

**Table 2:** Association between bevacizumab and patient survival

Variable		Survival Time, Months	HR^a^	95% CI	Pv	Adjusted HR^b^	95% CI	Pv
**Survival From Diagnosis**	No Bev	15.8(11.3–19.0)	Reference			Reference		
	Bev	16.0(12.5–23.0)	0.93	0.57–1.54	0.785	0.58	0.35–0.98	**0.043**
**Survival From 1st Recurrence**	No Bev	4.8(3.3–9.3)	Reference			Reference		
	Bev	9.0(6.0–12.0)	0.57	0.34–0.94	**0.028**	0.57	0.34–0.96	**0.033**

Note: Boldface type indicates *p* < 0.05

Bev = Bevacizumab; CI = Confidence Interval; HR = Hazard Ratio; Pv = *P*-value.

Median (Inter-quartile Range)

^a^ Univariate Cox regression.

^b^ Multivariable Cox regression, adjusted to time from diagnosis until 1st recurrence

**Fig. 3:**
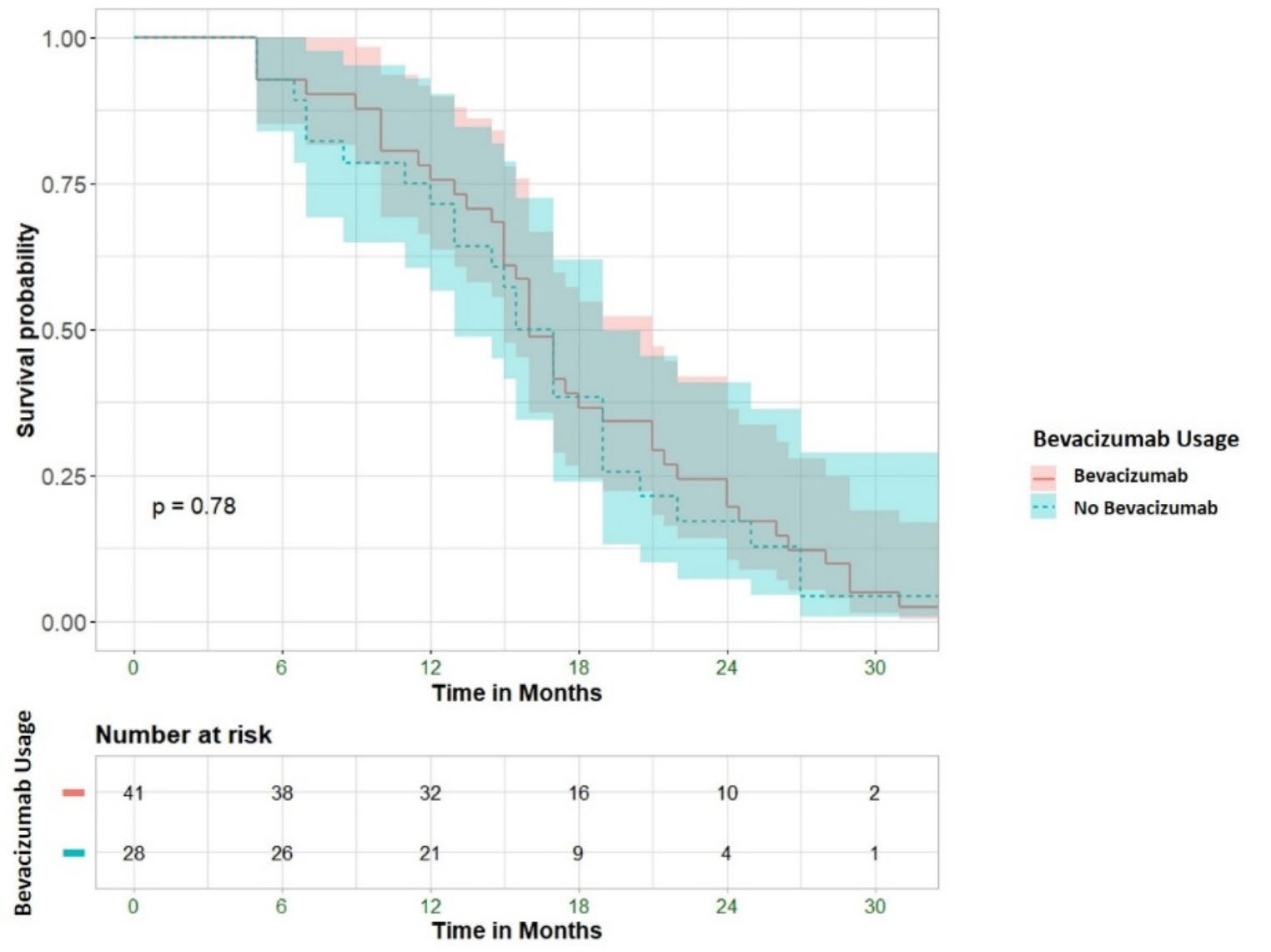
Overall survival from diagnosis. The lines represent the treatment group: blue = no Bevacizumab, red = Bevacizumab. The shades represent the 95% confidence interval. *P*-value from Log-Rank test

In general, bevacizumab as second-line treatment does not affect OS in SVZ GBM (Fig. 3).

**Fig. 4:**
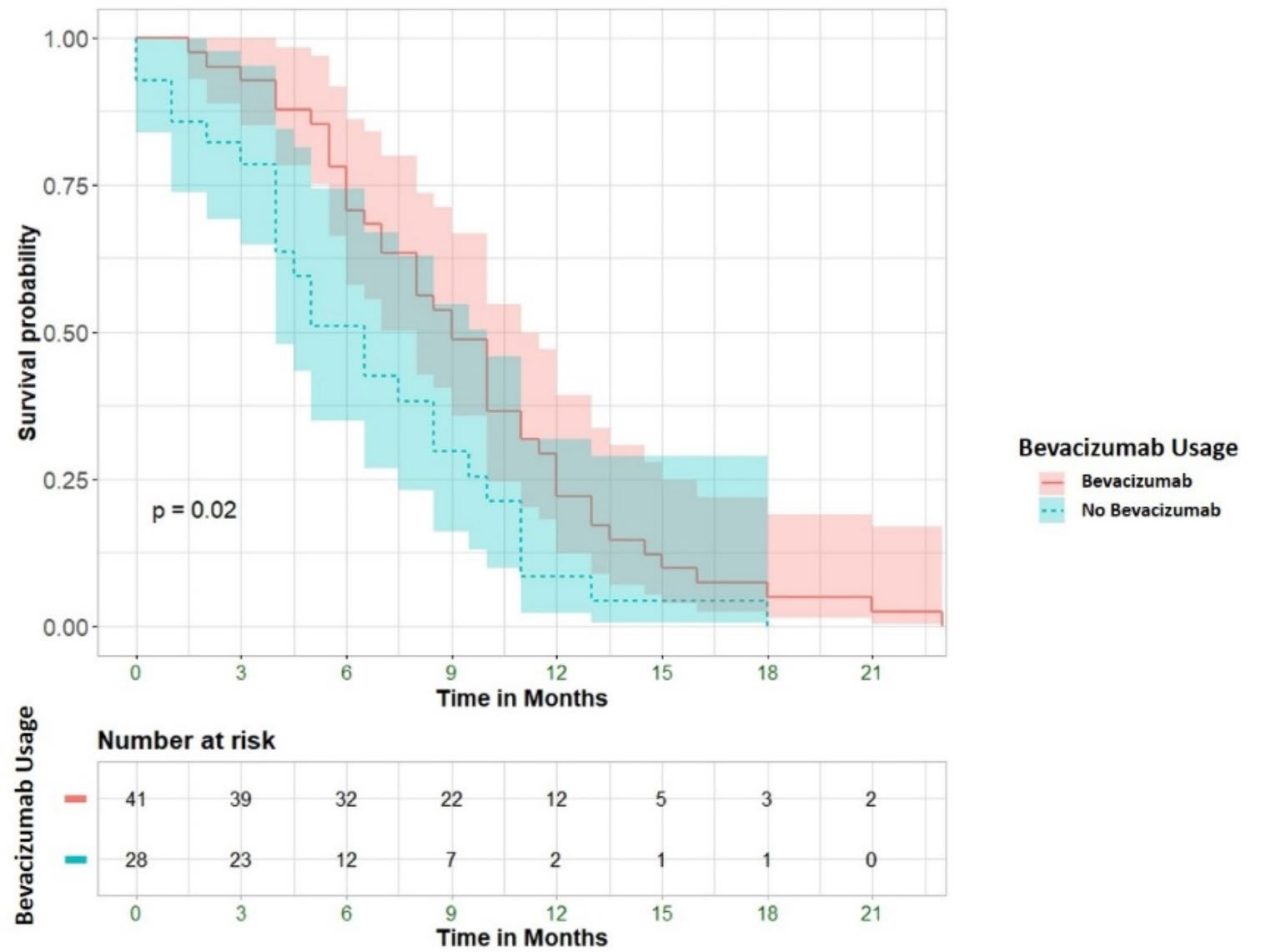
Overall survival from 1st recurrence. The lines represent the treatment group: blue = no Bevacizumab, red = Bevacizumab. The shades represent the 95% confidence interval. *P*-value from Log-Rank test

However, it significantly prolongs survival time from 1st relapse by an average of more than 4 months (HR = 0.57, 95% CI 0.34–0.94, *p* = 0.028) (Fig. 4).

This difference remains significant after adjusting for recurrent surgery (survival time from 1st relapse: HR = 0.57, 95%CI = 0.34–0.97, *p* = 0.038) and for age (survival time from 1st relapse: HR = 0.45, 95%CI = 0.24–0.84, *p* = 0.012). Furthermore, on a multivariate cox-regression analysis, when adjusting to time from diagnosis to 1st relapse (i.e., progression free survival), treatment with bevacizumab was associated with both prolonged OS as well as with prolonged survival time from 1st relapse (HR = 0.58 and 0.57, respectively; *p* = 0.043 and *p* = 0.033).

This significant association between bevacizumab treatment and prolonged survival from 1st relapse was also demonstrated when eliminating all patients (*n* = 23) with a combined second line therapy (8.65 ± 4.37 months vs. 5.71 ± 4.84 months, respectively; *p* = 0.044), reducing the potential favorable impact from other treatment modalities.

In a subgroup analysis, only patients with recurrent surgical resection of their relapsing tumor were included (*n* = 16). We than compared patients who were treated with a combined approach of re-surgery and bevacizumab (*n* = 6) to patients who were not treated with bevacizumab (*n* = 10). In this small subgroup the impact on OS was in favor of patients with the combined treatment of re-surgery + bevacizumab, reaching near significance (22.16 ± 7.81 m vs. 13.60 ± 6.86, *p* = 0.049; HR = 0.361 95%CI 0.108–1.209, *p* = 0.085). This combined treatment group of re-surgery + bevacizumab had the longest mean OS of the entire cohort. Similar to the primary analysis, the group treated with bevacizumab also had a prolonged survival time from 1st recurrence (Table 3).

**Table 3:** Association between Bevacizumab and Patient Survival according to Re-surgery Status

Variable		Re-Surgery			No Re-Surgery		
		Mean Survival Time, Months	95% CI	Pv	Mean Survival Time, Months	95% CI	Pv
**Survival From Diagnosis**	No Bev	14.1	9.4–18.8	*0.082*	17.7	14.2–21.3	0.584
	Bev	*22.2*	15.9–28.4		16.7	14.4–19.1	
**Survival From 1st Recurrence**	No Bev	6.7	4.0-9.4	0.334	6.5	4.2–8.8	**0.043**
	Bev	9.0	5.4–12.6		9.6	8.0-11.3	

Note: Boldface type indicates *p* < 0.05; Italic type indicated near significance

Bev = Bevacizumab; CI = Confidence Interval; HR = Hazard Ratio; Pv = *P*-value.

Log-Rank Test

In the subgroup of patients who did not undergo recurrent surgical resection (*n* = 51), no difference was found in OS between patients with and without bevacizumab. However, patients at this group that were treated with bevacizumab still had a significantly longer mean survival time from 1st recurrence (9.6 m vs. 6.5 m, *p* = 0.043; HR = 0.553 95%CI 0.302–1.016, *p* = 0.052).

Lastly, the beneficial impact on survival time from 1st recurrence was unique to patients receiving bevacizumab. When comparing patients who were treated with TTFields after recurrence(*n* = 13) to patients without this treatment (*n* = 54), no significant differences were found in OS nor in survival time from 1st recurrence (18.96 ± 5.89 vs. 16.04 ± 7.86, *p* = 0.142 and 9.23 ± 4.38 vs. 8.18 ± 5.46, *p* = 0.519; respectively).

## Discussion

Bevacizumab is FDA approved for recurrent GBM. Thus, it is rarely used as first line treatment and is not part of any updated protocol for newly diagnosed cases. As a result, most recent data on clinical benefits of bevacizumab is limited to its use as second line therapy. Our current study shows that for the unique subgroup of SVZ GBM, bevacizumab may favorably impact survival time from 1st relapse when given as second line treatment. SVZ GBM patients that were treated with bevacizumab had significantly prolonged survival from first relapse when compared to patients without such treatment. This difference remained significant in different subgroups, as long as the patients have received bevacizumab. This impact on survival was not demonstrated for any of the other second line treatment options. The studied cohort was homogenous for the majority of factors, with the exception of the variables recurrent surgery (significant) and age at diagnosis (near significant). Importantly, the observed association between bevacizumab treatment and prolonged survival from 1st relapse remained significant even after adjusting for these two variables.

Furthermore, we were able to show significant impact on OS as well. First, we have noticed that PFS (from diagnosis) was shorter in the subgroup of patients that eventually received bevacizumab following progression. The reason for that is not clear. Perhaps we proposed bevacizumab more easily to symptomatic patients with a rapid progression growth, and we were more inclined to propose re-surgery or a temozolomide rechallenge for patients with a longer interval time from diagnosis and first line therapy. This may also explain the higher rate of recurrent surgeries in the non-bevacizumab group. Theoretically, it may also mean that due to unidentified factors, the initial disease in the bevacizumab group was somehow more aggressive, leading to faster relapse. Importantly, most patients in the non-bevacizumab group were not denied the opportunity for bevacizumab therapy. Although they had a tendency toward lower KPS at progression, this difference was not significant and probably was not the reason that bevacizumab was not given eventually. In addition, bevacizumab was planned for more than half of them, but was not given or stopped prematurely due to patient’s preference, side effects or death. In any case, in order to eliminate this potential impact on OS, a multivariate cox regression analysis was performed, adjusting the groups for PFS. This resulted in significantly longer OS in patients that were treated with bevacizumab.

In addition, in the small subgroup of patients that underwent second surgery following relapse, those that were treated with bevacizumab following re-surgery had significantly longer OS, when compared to patients that did not receive bevacizumab. In fact, this group of patients, with a combined therapy of re-surgery and bevacizumab, had an impressive mean OS of 22.16 ± 7.81 m. Given that the published median OS of SVZ GBM patients is 7.8–11 months [1, 8, 9], this is a substantial improvement.

### Antiangiogenic therapy in GBM

Important feature of GBM is the vigorous and abnormal angiogenesis leading to disorganized and leaky blood vessels that is predominantly induced by the substantial elevation of VEGF activity, produced by tumor cells [14]. Great hopes were associated initially with anti – angiogenic therapy in GBM and the favorable impact of bevacizumab on PFS in GBM cases is well documented [27]. However, this needs to be interpreted with caution as these are mainly radiographic effects, secondary to decreased vascular permeability, while true tumor improvement is only marginal [28]. Bevacizumab did not prolong overall survival (OS) in patients with newly diagnosed [17, 18] or recurrent [16] GBM in phase 3 clinical trials. Other phase 2 trials have investigated bevacizumab in combination with several drugs, but none has displayed a significant impact on OS [29, 30]. Nevertheless, despite its limitations, bevacizumab remains the most commonly used anti-angiogenic agent in the treatment of recurrent GBM due to its role in reducing brain edema and symptomatic radiation brain necrosis [31].

Importantly, in all these studies [27, 32–36], groups were not categorized based on anatomical location and none of these trials have studied SVZ GBM separately, as a distinctive subgroup of GBM.

The main issue with the anti-angiogenic therapies is the lack of biomarkers and angiogenic profiles which allow identifying patients who may benefit from this kind of treatment [37]. A post hoc analysis of the ARTE trial has shown a survival benefit from the addition of bevacizumab to radiotherapy in comparison with radiotherpay alone in elderly patients with newly diagnosed GBM. This effect was depended on the presence of large contrast-enhancing lesions [19]. In another study, patients with evidence of enhanced tumor blood perfusion had a longer survival benefit with bevacizumab than those without vascular changes [38]. Thus, for specific, yet un- fully identified subgroups of GBM patients, anti-angiogentic treatment may carry true survival benefits.

In 2017, the FDA granted full approval for bevacizumab treatment of recurrent GBM, based on a phase 3, randomized study by Wick et al. [16]. While this study failed to show a significant increase in survival from recurrence with a bevacizumab-based treatment, progression-free survival from recurrence was significantly prolonged compared to chemotherapy alone. In that study, median survival from recurrence was 9.1 months in the bevacizumab treated group. Our results are in accordance with that, showing a median survival from recurrence of 9.0 months, which was significantly longer compared to the non- bevacizumab treated group. Our study however, albeit retrospective in design, shows more promising results. First, it shows that the impact on survival from recurrence was significant even when bevacizumab was used as monotherapy (and not necessarily when combined with chemotherapy or other treatment modality). Second, when adjusting to PFS from diagnosis and when considering a combined strategy of recurrent surgery + bevacizumab, bevacizumab treatment was associated with significantly longer OS (i.e., survival from diagnosis). To the best of our knowledge, such a potential impact on survival by an anti - angiogenic drug was not demonstrated before in cases of SVZ GBM.

### SVZ, glioblastoma stem cells and angiogenesis

The SVZ is a 3–5 mm layer between the lateral ventricle, corpus callosum, and striatum that harbors the largest population of NSCs in the brain [39]. Since NSCs are a core component of the SVZ, their presence has been considered to be responsible for the adverse prognosis of SVZ GBM [40]. The SVZ NSCs have demonstrated similar molecular profiles and share several distinctive characteristics with proliferative glioblastoma stem cells (GSCs) [41]. Genomic and proteomic studies comparing the SVZ and GBM support the hypothesis that the tumor stem cells and SVZ cells are related [3] and that GBM develops from NSCs in the SVZ [4].

The SVZ niche is believed to serve as a GSCs reservoir which contributes to resistance to therapy. Dalemans et al. have found that in SVZ GBM, tissue samples within the SVZ showed enrichment of gene sets involved in angiogenesis and hypoxia, compared to the samples outside of the SVZ region from the same tumors [42]. GSCs closely interact with the vascular niche of GBM and promote angiogenesis, mostly through the release of VEGF [6, 43]. Nearly two decades ago, Bao et al. have shown that tumors derived from GSCs were morphologically distinguishable from non-GSCs derived tumor populations by widespread tumor angiogenesis, necrosis, and hemorrhage. In addition, GSCs-derived population consistently secreted markedly elevated levels of VEGF [5]. GSCs can also directly participate in GBM vessel formation by transdifferentiating into endothelial cells or pericytes, the mural support cells of the microvasculature [44].

In a meta-analysis, increased radiation dose to the ipsilateral SVZ significantly increased PFS in GBM [45]. In addition, irradiation of NSCs was associated with better prognosis in patients with GBM contacting the SVZ [46]. Together, these data point to promising evidence that links tailored therapy of areas of the SVZ to increased measures of survival and highlight the importance of studying GBM in the context of the SVZ [3].

### Future directions

The theoretically unique impact of an anti-angiogenic therapy on survival in SVZ GBM cases may be related to the special role of the SVZ as the largest neural stem cell niche in the adult brain and its possible impact on angiogenesis. However, much more elaborated research is needed in order to prove such correlation. Secondly, further research is needed in order to accurately define the subpopulation of patients that will benefit most from combined therapy such as an increased radiation dose to the SVZ + re-surgery + anti angiogenic therapy. Lastly, we offer to consider clinical trials on the use of anti-angiogenic therapy as first line treatment in SVZ GBM cases.

### Study limitations

The study was limited by its retrospective design which harbors inherent biases. We could not account for the effect of the experience gained by staff and surgeons over the 10-year period of the study in terms of surgical outcomes and prognostic parameters. In addition, although the basic adjuvant chemoradiation protocol did not change during the study period, several other treatments were added, especially as a second line. Although we know in retrospect that not all of them had a meaningful impact on survival, their use interfered with the homogeneity of the cohort. Some statistical analyses were limited by the cohort’s size, especially when comparing more homogenous yet smaller subgroups. These sub-analyses should be considered hypothesis-generating as solid conclusions cannot be made from such small cohorts and their survival outcomes should be considered with caution. In addition, small cohort has narrowed our ability to perform a propensity score matching, which would have shrinkage our cohort further. Nevertheless, it should be emphasized that the lack of major differences for most variables between the groups has substantially reduced the need for a propensity matching. A s for other studies on tumor’s progression, a major limitation is the lack of pathology – proven recurrence in many cases. Although we have defined progression based on RANO criteria for progression in high grade gliomas, it is still possible that some cases were pseudo-progression. Nevertheless, this limitation should not significantly change our observation on the potential survival impact of bevacizumab in SVZ GBM. Lastly, this study was limited to the unique group of SVZ GBM. We have not studied the prognostic effects of bevacizumab in non-SVZ GBM cases. Although level 1 evidence shows no positive association between bevacizumab administration and improved survival in GBM cases in general, including at relapse, no specific data exists on non-SVZ GBM cases. This is a potential for future studies.

## Conclusion

SVZ GBM are increasingly recognized as a distinct group of high-grade gliomas, with characterized radiological, molecular, clinical and prognostic features. Our current study supports this newly observations by showing that, in contradiction to current data, the use of bevacizumab as second line therapy in SVZ GBM cases may favorably impact survival from relapse. This impact remained significant even when bevacizumab was given as a monotherapy. Additionally, in certain yet-to-be-identified sub-populations, bevacizumab may extend overall survival. There is a potential favorable synergetic effect of recurrent surgery with bevacizumab therapy, as in the subgroup of patients who did not undergo recurrent surgical resection, no difference was found in OS between patients with and without bevacizumab.

## Data Availability

No datasets were generated or analysed during the current study.

## References

[1] Berendsen S, van Bodegraven E, Seute T et al (2019) Adverse prognosis of glioblastoma contacting the subventricular zone: Biological correlates. PLoS ONE 14:e0222717 31603915 10.1371/journal.pone.0222717 PMC6788733

[2] Woo P, Ho J, Lam S et al (2018) A comparative analysis of the usefulness of Survival Prediction models for patients with Glioblastoma in the Temozolomide era: the importance of Methylguanine Methyltransferase Promoter Methylation, extent of Resection, and Subventricular Zone Location. World Neurosurg 115:e375–e385 29678708 10.1016/j.wneu.2018.04.059

[3] Beiriger J, Habib A, Jovanovich N et al (2022) The Subventricular Zone in Glioblastoma: Genesis, maintenance, and modeling. Front Oncol 12:790976 35359410 10.3389/fonc.2022.790976 PMC8960165

[4] Lee JH, Lee JE, Kahng JY et al (2018) Human glioblastoma arises from subventricular zone cells with low-level driver mutations. Nature 560:243–247 30069053 10.1038/s41586-018-0389-3

[5] Bao S, Wu Q, Sathornsumetee S et al (2006) Stem cell-like glioma cells promote tumor angiogenesis through vascular endothelial growth factor. Cancer Res 66:7843–7848 16912155 10.1158/0008-5472.CAN-06-1010

[6] D’Alessio A, Proietti G, Lama G et al (2016) Analysis of angiogenesis related factors in glioblastoma, peritumoral tissue and their derived cancer stem cells. Oncotarget 7:78541–78556 27705944 10.18632/oncotarget.12398 PMC5346658

[7] Mistry AM (2019) Clinical correlates of subventricular zone-contacting glioblastomas: a meta-analysis. J Neurosurg Sci 63:581–587 29205011 10.23736/S0390-5616.17.04274-6

[8] Mistry AM, Dewan MC, White-Dzuro GA et al (2017) Decreased survival in glioblastomas is specific to contact with the ventricular-subventricular zone, not subgranular zone or corpus callosum. J Neurooncol 132:341–349 28074322 10.1007/s11060-017-2374-3 PMC5771712

[9] Comas S, Luguera E, Molero J et al (2021) Influence of glioblastoma contact with the subventricular zone on survival and recurrence patterns. Clin Transl Oncol 23:554–564 32728970 10.1007/s12094-020-02448-x

[10] Daniele Armocidaa AP, Mauro Palmieria GD, Andread M, Salvatib A, Santoroa A, Frati (2021) Periventricular Zone involvement as a predictor of survival in glioblastoma patients: a single centre cohort-comparison investigation concerning a distinct clinical entity, vol 25. Advanced Techniques and Case Management, Interdisciplinary Neurosurgery

[11] Jafri NF, Clarke JL, Weinberg V, Barani IJ, Cha S (2013) Relationship of glioblastoma multiforme to the subventricular zone is associated with survival. Neuro Oncol 15:91–96 23095230 10.1093/neuonc/nos268 PMC3534420

[12] Pellerino A, Bruno F, Soffietti R, Rudà R (2023) Antiangiogenic therapy for malignant brain tumors: does it still Matter? Curr Oncol Rep 25:777–785 37071295 10.1007/s11912-023-01417-1 PMC10256654

[13] Kazazi-Hyseni F, Beijnen JH, Schellens JH, Bevacizumab (2010) Oncologist 15:819–825 10.1634/theoncologist.2009-0317 PMC3228024 20688807

[14] Rajaratnam V, Islam MM, Yang M, Slaby R, Ramirez HM, Mirza SP (2020) Glioblastoma: Pathogenesis and current status of Chemotherapy and other Novel treatments. Cancers (Basel) 12 10.3390/cancers12040937 PMC7226351 32290213

[15] Narayana A, Gruber D, Kunnakkat S et al (2012) A clinical trial of bevacizumab, temozolomide, and radiation for newly diagnosed glioblastoma. J Neurosurg 116:341–345 22035272 10.3171/2011.9.JNS11656

[16] Wick W, Gorlia T, Bendszus M et al (2017) Lomustine and Bevacizumab in Progressive Glioblastoma. N Engl J Med 377:1954–1963 29141164 10.1056/NEJMoa1707358

[17] Chinot OL, Wick W, Mason W et al (2014) Bevacizumab plus radiotherapy-temozolomide for newly diagnosed glioblastoma. N Engl J Med 370:709–722 24552318 10.1056/NEJMoa1308345

[18] Gilbert MR, Dignam JJ, Armstrong TS et al (2014) A randomized trial of bevacizumab for newly diagnosed glioblastoma. N Engl J Med 370:699–708 24552317 10.1056/NEJMoa1308573 PMC4201043

[19] Wirsching HG, Roelcke U, Weller J et al (2021) MRI and (18)FET-PET predict Survival Benefit from Bevacizumab Plus Radiotherapy in patients with isocitrate dehydrogenase wild-type Glioblastoma: results from the Randomized ARTE Trial. Clin Cancer Res 27:179–188 32967939 10.1158/1078-0432.CCR-20-2096

[20] Louis DN, Ohgaki H, Wiestler OD et al (2007) The 2007 WHO classification of tumours of the central nervous system. Acta Neuropathol 114:97–109 17618441 10.1007/s00401-007-0243-4 PMC1929165

[21] Louis DN, Perry A, Reifenberger G et al (2016) The 2016 World Health Organization Classification of Tumors of the Central Nervous System: a summary. Acta Neuropathol 131:803–820 27157931 10.1007/s00401-016-1545-1

[22] Louis DN, Perry A, Wesseling P et al (2021) The 2021 WHO classification of tumors of the Central Nervous System: a summary. Neuro Oncol 23:1231–1251 34185076 10.1093/neuonc/noab106 PMC8328013

[23] Stupp R, Mason WP, van den Bent MJ et al (2005) Radiotherapy plus concomitant and adjuvant temozolomide for glioblastoma. N Engl J Med 352:987–996 15758009 10.1056/NEJMoa043330

[24] Perry JR, Laperriere N, O’Callaghan CJ et al (2017) Short-course Radiation plus Temozolomide in Elderly patients with Glioblastoma. N Engl J Med 376:1027–1037 28296618 10.1056/NEJMoa1611977

[25] Wen PY, Macdonald DR, Reardon DA et al (2010) Updated response assessment criteria for high-grade gliomas: response assessment in neuro-oncology working group. J Clin Oncol 28:1963–1972 20231676 10.1200/JCO.2009.26.3541

[26] Karschnia P, Young JS, Dono A et al (2023) Prognostic validation of a new classification system for extent of resection in glioblastoma: a report of the RANO resect group. Neuro Oncol 25:940–954 35961053 10.1093/neuonc/noac193 PMC10158281

[27] Ameratunga M, Pavlakis N, Wheeler H, Grant R, Simes J, Khasraw M (2018) Anti-angiogenic therapy for high-grade glioma. Cochrane Database Syst Rev 11:Cd008218 30480778 10.1002/14651858.CD008218.pub4 PMC6516839

[28] Zikou A, Sioka C, Alexiou GA, Fotopoulos A, Voulgaris S, Argyropoulou MI (2018) Radiation Necrosis, Pseudoprogression, Pseudoresponse, and Tumor Recurrence: Imaging Challenges for the Evaluation of Treated Gliomas. Contrast Media Mol Imaging 2018:6828396 10.1155/2018/6828396 PMC6305027 30627060

[29] Gilbert MR, Pugh SL, Aldape K et al (2017) NRG oncology RTOG 0625: a randomized phase II trial of bevacizumab with either irinotecan or dose-dense temozolomide in recurrent glioblastoma. J Neurooncol 131:193–199 27770279 10.1007/s11060-016-2288-5 PMC5263144

[30] Raizer JJ, Giglio P, Hu J et al (2016) A phase II study of bevacizumab and erlotinib after radiation and temozolomide in MGMT unmethylated GBM patients. J Neurooncol 126:185–192 26476729 10.1007/s11060-015-1958-z PMC4826294

[31] Zhuang H, Shi S, Yuan Z, Chang JY (2019) Bevacizumab treatment for radiation brain necrosis: mechanism, efficacy and issues. Mol Cancer 18:21 30732625 10.1186/s12943-019-0950-1 PMC6367784

[32] Balana C, De Las Penas R, Sepúlveda JM et al (2016) Bevacizumab and temozolomide versus temozolomide alone as neoadjuvant treatment in unresected glioblastoma: the GENOM 009 randomized phase II trial. J Neurooncol 127:569–579 26847813 10.1007/s11060-016-2065-5

[33] Herrlinger U, Schäfer N, Steinbach JP et al (2016) Bevacizumab Plus Irinotecan Versus Temozolomide in newly diagnosed O6-Methylguanine-DNA methyltransferase nonmethylated Glioblastoma: the Randomized GLARIUS Trial. J Clin Oncol 34:1611–1619 26976423 10.1200/JCO.2015.63.4691

[34] Lee EQ, Kaley TJ, Duda DG et al (2015) A Multicenter, Phase II, Randomized, Noncomparative Clinical Trial of Radiation and Temozolomide with or without Vandetanib in newly diagnosed Glioblastoma patients. Clin Cancer Res 21:3610–3618 25910950 10.1158/1078-0432.CCR-14-3220 PMC4790106

[35] Nabors LB, Fink KL, Mikkelsen T et al (2015) Two cilengitide regimens in combination with standard treatment for patients with newly diagnosed glioblastoma and unmethylated MGMT gene promoter: results of the open-label, controlled, randomized phase II CORE study. Neuro Oncol 17:708–717 25762461 10.1093/neuonc/nou356 PMC4482861

[36] Stupp R, Hegi ME, Gorlia T et al (2014) Cilengitide combined with standard treatment for patients with newly diagnosed glioblastoma with methylated MGMT promoter (CENTRIC EORTC 26071– 22072 study): a multicentre, randomised, open-label, phase 3 trial. Lancet Oncol 15:1100–1108 25163906 10.1016/S1470-2045(14)70379-1

[37] Gil-Gil MJ, Mesia C, Rey M, Bruna J (2013) Bevacizumab for the treatment of glioblastoma. Clin Med Insights Oncol 7:123–135 23843722 10.4137/CMO.S8503 PMC3682734

[38] Lu-Emerson C, Duda DG, Emblem KE et al (2015) Lessons from anti-vascular endothelial growth factor and anti-vascular endothelial growth factor receptor trials in patients with glioblastoma. J Clin Oncol 33:1197–1213 25713439 10.1200/JCO.2014.55.9575 PMC4517055

[39] Doetsch F, Caillé I, Lim DA, García-Verdugo JM, Alvarez-Buylla A (1999) Subventricular zone astrocytes are neural stem cells in the adult mammalian brain. Cell 97:703–716 10380923 10.1016/s0092-8674(00)80783-7

[40] Spiteri I, Caravagna G, Cresswell GD et al (2019) Evolutionary dynamics of residual disease in human glioblastoma. Ann Oncol 30:456–463 30452544 10.1093/annonc/mdy506 PMC6442656

[41] Matarredona ER, Pastor AM (2019) Neural stem cells of the Subventricular Zone as the origin of human glioblastoma stem cells. Therapeutic Implications Front Oncol 9:779 31482066 10.3389/fonc.2019.00779 PMC6710355

[42] Dalemans DJZ, Berendsen S, Draaisma K, Robe PA, Snijders TJ (2021) Glioblastomas within the Subventricular Zone Are Region-Specific enriched for mesenchymal transition markers: an Intratumoral Gene expression analysis. Cancers (Basel) 13 10.3390/cancers13153764 PMC8345101 34359668

[43] Oka N, Soeda A, Inagaki A et al (2007) VEGF promotes tumorigenesis and angiogenesis of human glioblastoma stem cells. Biochem Biophys Res Commun 360:553–559 17618600 10.1016/j.bbrc.2007.06.094

[44] Cheng L, Huang Z, Zhou W et al (2013) Glioblastoma stem cells generate vascular pericytes to support vessel function and tumor growth. Cell 153:139–152 23540695 10.1016/j.cell.2013.02.021 PMC3638263

[45] Şuşman S, Leucuţa DC, Kacso G, Florian ŞI (2019) High dose vs low dose irradiation of the subventricular zone in patients with glioblastoma-a systematic review and meta-analysis. Cancer Manag Res 11:6741–6753 31410064 10.2147/CMAR.S206033 PMC6645358

[46] Kahng JY, Kang BH, Lee ST et al (2023) Clinicogenetic characteristics and the effect of radiation on the neural stem cell niche in subventricular zone-contacting glioblastoma. Radiother Oncol 186:109800 37423479 10.1016/j.radonc.2023.109800

